# Academic word coverage and language difficulty of reading passages in College English Test and Test of English for Academic Purposes in China

**DOI:** 10.3389/fpsyg.2023.1171227

**Published:** 2023-06-30

**Authors:** Huidan Liu, Xiaomeng Shi, Jing Qiu, Ying Shi, Yunhan Hao, Liya Zhu, Chengjie Yan, Huadong Li

**Affiliations:** College of Foreign Languages, Shanghai Maritime University, Shanghai, China

**Keywords:** College English Test (CET), Test of English for Academic Purposes (TEAP), academic word coverage, language difficulty, Flesch–Kincaid Grade Level, English for Academic Purposes (EAP), academic literacy

## Abstract

The field of English for Specific Purposes (ESP) has gained considerable attention, mainly due to the growing importance of English teaching and the need for preparing international professionals. The primary academic emphasis of this field is largely centered on the study of English for Academic Purposes (EAP). It has become increasingly important for China to provide EAP education to its vast population of college students, who represent a significant proportion of global tertiary-level English learners. By doing so, they can improve their academic literacy and participate more effectively in international academic communication. In 2016, China’s College English Test Examination Board issued the *National College English Test Syllabus* which emphasized the importance of academic English literacy in the test design. This syllabus was released during a contentious debate among Chinese academics about the role of EAP in college English teaching and testing. Despite this heated discussion, there are currently few studies that have analyzed the linguistic nature of academic English tests in a quantitative manner. This paper calculates the academic word coverage of the reading passages in two crucial English proficiency tests designed for college students in China, the College English Test (CET) and the Test of English for Academic Purposes (TEAP). It is found that the academic word coverage of CET is increasing from the year 2013 to 2021 and that the academic word coverage of TEAP is slightly higher than that of CET. In this sense, these English tests can meet the requirements for measuring Chinese college students’ academic literacy. It is also found that there is a positive correlation between academic word coverage and language difficulty as is indicated by Flesch–Kincaid Grade Level. These findings provide an empirical reference for the study of academic English tests in China, and other parts of the world, and contribute to EAP teaching and testing reform for the development of students’ academic literacy.

## 1. Introduction

Since the 21st century, college English^1^ teaching and testing in China have undergone continuous reform and development. It was in the year 2013 that Shanghai Municipal Education Commission issued *A Framework of Reference for English as a Foreign Language Teaching at Tertiary Level in Shanghai (Trial Implementation)* (abbreviated as *Framework* hereafter) (Shanghai Foreign Languages Teaching Advisory Board, 2013). The *Framework* aimed to promote students’ communication skills in academic English and prepare students for a career in their professional fields worldwide. The *Framework* stipulated that English for Academic Purposes (EAP) should replace English for General Purposes (EGP) and become the core content of college English teaching in institutions of higher learning in Shanghai. In 2015 and 2020, another government document about college English teaching, *Guidelines on College English Teaching* (*Guidelines* hereafter) also emphasized the importance of academic English teaching (The National Foreign Languages Teaching Advisory Board under the Ministry of Education, 2015, 2020). Issued by the Ministry of Education of the People’s Republic of China, the *Guidelines* specified that EAP should be included in college English teaching and testing. Until now, multiple EAP courses have been offered in many colleges and universities in China. However, there is a hot debate over how much EAP courses account for the college English curricula in China.

Many scholars insist that EGP be the core in college English teaching. For example, Wang and Yao (2013) and Wang and Wang (2019) argued that EGP should take the primary part and that EAP can only be taught in top universities in China where students in general have a better command of the English language. Hu and Xie (2014) predicted that EGP shall be the main dishes of college English teaching in China now and in the near future, and that EAP shall be the side dishes.

EGP has long dominated the teaching of English as a Foreign Language (EFL) in China, and most students have been learning EGP when they are receiving English education in primary school, middle school, and high school. Moreover, in China’s higher education, EGP courses have been compulsory for non-English majors since the 1980s (Gao and Bartlett, 2014), covering “campus life, personal growth, politeness, appreciation of music, health and hygiene, friendship and human emotions, paths to success, and cultural values” (Cheng, 2016, p. 213). Under this circumstance, some scholars proposed that college English should be reformed and different from what students have been learning before college. To be specific, EAP shall be the primary, even the core content of college English in China, in order to develop students’ academic literacy. Qin (2003) and Zhang (2003) argued that the mainstream of college English should focus on ESP related to the students’ majors. Yang (2018) also believed that academic English teaching would become the mainstream of college English teaching. Cai (2015, 2022) further proposed that college English teaching should be targeted at ESP teaching and that EAP courses should be set as compulsory courses because college students use English mainly for academic purposes rather than for general purposes. Liang and Wang (2020) seemed to be eclectic and suggested that EAP should be integrated into college English instead of being a substitution of, or a side dish to, EGP.

EAP advocates, including Cai (2015, 2022), asserted that the relevance of EAP lies in its direct link to the purpose for which English is used, rather than its level of language difficulty. However, other scholars, such as Wang and Yao (2013) and Wang and Wang (2019), argued that the English proficiency of Chinese university students varies greatly, with many still at a low level. Therefore, they recommend that these students learn EGP instead of EAP. This argument is based on an implied assumption that academic language and language difficulty are positively related.

To test the above assumption, one of the essential areas that we can delve into is English language testing. Testing is an indispensable part of language teaching. Meanwhile, it is also supposed to mirror the outcome of teaching. Theoretically speaking, the academic or non-academic nature of language testing can reflect the academic or non-academic features of the corresponding language teaching. To quantitatively examine the academic content of large-scale nationwide English testing in China in terms of academic vocabulary, this study employed the widely used Academic Word List (AWL) compiled by Coxhead (2000) to analyze the academic word coverage of China’s English tests at the tertiary level.

Specifically, this study focuses on two types of representative English proficiency tests in China, that is, College English Test (CET) and Test of English for Academic Purposes (TEAP). This study employs corpus tools to examine the AWL coverage of the reading passages in the two types of tests to describe the academic features of these tests. The study attempts to answer the questions whether and to what extent CET and TEAP are EAP tests. The answer to these questions may help resolve the debate as was mentioned earlier. The study may have implications for the design of language testing aimed to develop students’ academic literacy both in China and in the international academic community.

In addition to probing into the academic vocabularies in the two types of English tests, this study also attempts to examine the content validity of the tests based on the text analysis. One way to analyze the content validity of a text is to calculate its readability and specialization. Readability refers to the ease with which a reader can understand a written text, which can be calculated and transferred into Flesch–Kincaid Grade Level (abbreviated as Grade Level) (Jiang et al., 2020; Wang, 2021), while specialization is estimated from the proportion of vocabulary closely related to students’ majors in reading passages in the tests (Dong, 2020). For a text of a given genre or type, there appears to be a relationship between language difficulty (the opposite of readability) and the specialization of the text. For an academic text, specialization (in the sense of how academic a text is in this study) can be examined via the parameter of AWL coverage.

To sum up, the present study attempts to answer three questions:

1. Are CET and TEAP testing EAP? Furthermore, if they are, to what extent are they serving this purpose?
2. Do CET and TEAP fulfill the requirements specified in the *Guidelines* and the *Framework* in the sense of measuring test-takers academic literacy?
3. Does coverage of academic words correlate with language difficulty of the texts in CET and TEAP?

## 2. Literature review

### 2.1. English for Academic Purposes

**Figure 1:**
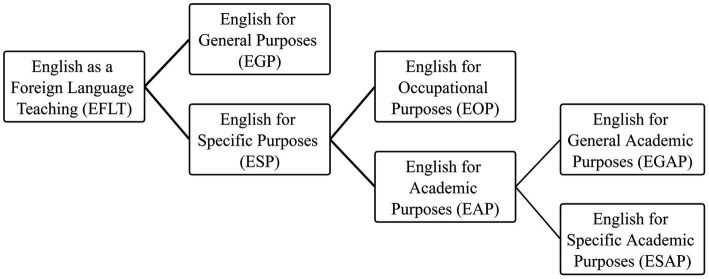
Classification of English as a Foreign Language Teaching.

The origin of the study of EAP can be traced back to the study of English as a Foreign Language Teaching (EFLT) (Hutchinson and Waters, 1987). EAP, as a branch of English for Specific Purposes (ESP), can be further divided into English for General Academic Purposes (EGAP) and English for Specific Academic Purposes (ESAP) (Jordan, 1997) (see Figure 1). As was suggested by the *Framework*, courses teaching EGAP shall be compulsory and account for at least 60% of the total credits in the curriculum of college English for non-English majors in Shanghai.

Many studies are focusing on the content of EAP teaching. Mu et al. (2015) proposed the importance of intercultural differences in metadiscourse of Chinese and English research articles. Gao and Cui (2021) conducted a three-year-long case study to trace an English teacher’s journey in EAP reform from the perspective of conceptual metaphor theory and identity research, hoping to “help push policymakers to open diversified education programs for preservice teachers to meet the new expectations of Chinese society better” (p. 9). Abdollahzadeh et al. (2022) explored the potential relationships between L2 motivation and transformative engagement in academic reading among undergraduate EAP learners and suggested that teachers, policymakers, and material and curricula developers should pay much attention to L2 motivation of students to engage them successfully in academic reading skill development. Moreover, Yapp et al. (2021) insisted that L2 reading strategy interventions should be extended to higher education institutions in the future. In brief, the EAP studies indicate that a higher proportion of EAP teaching in the curriculum contributes considerably to developing students’ academic literacy.

In addition to academic English teaching, many scholars have also frequently discussed testing related to ESP. Testing Language for Specific Purposes (TLSP), as a branch of language testing, evaluates students’ English competence after ESP learning. Students are tested in an original mission to demonstrate their linguistic ability regarding vocabulary and syntax required in specific areas (Douglas, 2000). Responding to the evolving emphasis on ESP in college English teaching Fu and Zhao (2017) proposed to establish an ESP test to propel the development of ESP teaching in China. It is reasonable to design tests for academic purposes because their construct validity implies that the corresponding EAP tests can evaluate the existing EAP courses (Cai, 2012). For example, the Pearson Test of English Academic (PTE Academic) initiated by Britain Pearson Education provides a detailed reference standard for students’ academic English competence (Wang, 2012). In a sense, EAP testing, as a response to EAP teaching, while testing students’ academic English proficiency, exerts a washback effect upon English teaching. According to Jin (2004), the washback effect means that language testing helps to improve language teaching. Therefore, EAP testing can also serve the purpose of developing students’ academic literacy.

At present, there is a lack of research, particularly in quantitative ways, that explores the academic features of China’s English tests. To fill this gap, the present study attempts to conduct a quantitative analysis of the use of academic words in the two representative English proficiency tests in China, CET and TEAP to provide empirical support for the practice of EAP teaching in China in recent decades.

### 2.2. Academic Word List

Academic Word List (AWL) proposed by Averil Coxhead (2000, 2011) was compiled based on a corpus of academic English texts including humanities, commerce, law, and science, with a total of 3.5 million words in 28 subject areas. It was compared and contrasted with the other corpus of novels with a comparable total of words. The AWL contains 570 word families. The coverage of academic words over the established academic English corpus is consistently around 10%, while the coverage of academic words in the corresponding fiction corpus, which is usually regarded as the texts of English for General Purposes, is merely 1.4%. Coxhead’s (2000) findings on the occurrence of AWL have been examined by other researchers who have demonstrated that AWL’s coverage in other academic English texts is approximately 10%. Hyland and Tse (2007) built a corpus of English texts from science, engineering, and social science, and found that the AWL coverage of these texts was 10.6%. In addition, Vongpumivitch et al. (2009) conducted a lexical study to explore the use of AWL in the periodical literature in the field of applied linguistics and found that AWL vocabulary accounted for 11.17% of the overall vocabulary in the corpus, higher than that in the humanities sub-corpus created by Coxhead (2000), that is, 9.3%, but both figures remain close to 10%. In addition, scholars have studied the AWL coverage in academic papers on medicine (Chen and Ge, 2007), agriculture (Martinez et al., 2009), and engineering sciences (Ward, 2009), and found that the AWL coverage of the texts focusing on these fields is around 10%. Statistically speaking, it appears that Coxhead’s (2000) AWL and its coverage of 10% are viable and effective tools for determining whether a text can be regarded as an academic text.

AWL has been applied to corpus-based research on EAP. Coxhead (2012) emphasized the importance of academic vocabulary for academic writing and suggested that EAP teachers should give advice about which readings and vocabulary need to be focused on to meet the needs of learners. Cons (2012) conducted a study on the utilization of academic vocabulary in evaluating students’ interpretive reading and analytical writing skills, and discovered that English language learners require greater exposure to academic words, especially in writing. However, Paribakht and Webb (2016) examined the AWL coverage in English proficiency tests used for admission purposes at Canadian universities and found that AWL coverage did not correlate with the overall reading and listening comprehension scores. However significant relationships were found between academic vocabulary use and essay scores in some text types (Csomay and Prades, 2018). As Masrai and Milton (2018, p. 44) stated that “the AWL has become central to the teaching of EAP,” Chinese students have used academic words to present their ideas concisely in oral English presentations (Cribb and Wang, 2019). Attempts were made to explore the relationship between AWL coverage and language difficulty of texts from English tests. By calculating the AWL coverage of college English textbooks in China, Li et al. (2019) and Li and Jiang (2022) found a strong correlation between AWL coverage and language difficulty of reading passages in college English textbooks in China.

### 2.3. Language testing

Based on language acquisition and language teaching, language testing is designed to measure whether students have achieved the language ability required by the language teaching syllabus or educational programs (Bachman, 1990). In China, College English Test (CET) has been designed as a “national standardized English proficiency test” (Wang and Yao, 2013, p. 12) issued by the Ministry of Education of the People’s Republic of China and National Education Examinations Authority in 1987. Over the years, it has been developed into “a large-scale English test that reaches the standards of international language testing, and this test also has great social credibility and international influence” (Yang, 2018, p. 31). Comprised of College English Test Band 4 (CET-4) and Band 6 (CET-6), CET has been carried out to measure test-takers English competence at different stages of English learning. CET-4 is designed for students to take in their fourth semester of college education and CET-6 is designed for students to take in their sixth semester.

According to the *National College English Test Syllabus* (*Syllabus* hereafter) issued in 2016 (China’s College English Test Examination Board, 2016), the requirements of language skills tested via CET correspond to the “basic requirements” and “intermediate requirements” set in the *Guidelines*. According to the *Guidelines*, to meet the “basic requirements,” Chinese college students not only need to acquire such knowledge as English pronunciation, vocabulary, grammar and text structure, but also to master another 2,000 words in addition to the vocabulary learned in the Chinese high school, of which 400 words are related to students’ academic study or future work. Students are expected to achieve a basic understanding of oral or written materials of medium language difficulty, involving common communication topics. To meet the “intermediate requirements,” a higher standard, Chinese college students also need to use such knowledge about English pronunciation, intonation, vocabulary, grammar, and text structure but in a more proficient way. The number of words they should master is increased to 3,000 words in addition to the vocabulary learned in the Chinese high school, of which 600 words are related to students’ academic study or future work. And students are expected to reach a better understanding of oral or written materials of medium language difficulty, of familiar materials or those related to specific majors and to have an understanding of the logical relationships, text structure and connotation of the materials (The National Foreign Languages Teaching Advisory Board under the Ministry of Education, 2020). These progressive teaching objectives set in the *Guidelines* lead to different requirements of language skills for CET-4 and CET-6 takers from “general” English to “academic” English.

In contrast, the design and implementation of EAP tests in China are still in the developing stage. The Test of English for Academic Purposes (TEAP), organized in China in 2015, is a standardized test initiated by China English Language Education Association and Shanghai Foreign Languages Teaching Advisory Board. Given the interdisciplinary integration of humanities and sciences, the academic vocabularies examined in this test were collected from the Academic English Proficiency Rating Scale and the General Academic English Teaching Reference Vocabulary proposed by the *Framework*. TEAP aims to examine whether non-English majors can meet the requirements set forth in the *Framework* and to improve the teaching of college English for academic purposes in light of the ongoing influence of language testing on classroom teaching. According to the *Framework* and the *Guidelines*, English tests at the tertiary level should be able to evaluate students’ academic literacy. Specifically, English testing for academic purposes aims to test whether students can understand English lectures, absorb academic information, write reviews and papers, and conduct academic exchanges in English (Cai, 2012). For example, the Test of English as a Foreign Language (TOEFL), as a typical EAP test proposed and promoted by the Educational Testing Service (ETS) in the United States, evaluates the academic English level of test-takers. Another two large-scale tests, the International English Language Testing System (IELTS) and Japan’s Test of English for Academic Purposes also serve a similar purpose.

Language testing partly reflects the language teaching system. Against the backdrop where EGP teaching seems to be the mainstream of China’s college English teaching, it is essential to measure whether and to what extent China’s CET tests have undergone some changes in terms of academic content design since the issuing of the *Guidelines* and the *Syllabus,* and whether the TEAP test has matched up with the academic goals set by the *Framework.* So the question remains to be answered: whether these tests fulfill the goals in the *Guidelines* and the *Framework* in order to exert influence upon students’ English learning, especially in the development of their academic literacy.

Validity is an essential parameter in developing language tests. It is a fundamental requirement for engineering and evaluating a language test (Jin, 2006; Xi and Zhang, 2020). Validity studies have been integrated into China’s college tests, such as CET (Liu and Gu, 2013; Jin and Yang, 2018) and Tests for English Major (TEM) (Zou, 2007). Validity is taken as the most crucial measure of test quality in researches conducted on large-scale standardized tests. For example, Yang and Weir (1998) conducted a three-year validity study to analyze the CET test’s content validity. Tang and Ma (2020) tried to optimize the design of the translation part of CET by studying its content validity. These studies verify that valid content contributes considerably to accomplishing the specific goals of a given test. Therefore, this study will examine the relationship between readability and coverage of academic words in the tests to discuss the validity of CET and TEAP and to find out whether the two types of tests have met the requirements set by the corresponding syllabi.

## 3. Materials and methods

The present study uses AWL coverage to measure the academic level of China’s College English Tests in recent years, including CET-4, CET-6, and TEAP. It further explores the correlation between AWL coverage and language difficulty. Language difficulty is evaluated via Grade Level in this paper.

### 3.1. Materials

This study builds three corpora comprised of the reading passages in CET-4, CET-6, and TEAP. As the *Framework* was issued in 2013 to call for academic reform in college English teaching, the time range of CET test papers examined in this study is from December 2013 to December 2021. It is anticipated that this setting of the time range will investigate any possible alterations in the CET during these years in the sense of academic words. This research, in particular, has chosen to examine Section A, Section B, and Section C of the Reading Comprehension component in all CET-4 and CET-6 papers, and has eliminated all related questions in these three sections. The corpus of CET-4 is comprised of 204 reading passages from 51 test papers, with a total of 107,386 tokens; the corpus of CET-6 is also comprised of 204 reading passages from 51 test papers, with 127,143 tokens (see Table 1).

**Table 1:** The corpora of CET-4 and CET-6.

Test	Test papers	Reading passages	Running words
CET-4	51	204	107,386
CET-6	51	204	127,143

The corpus of TEAP is from one sample test paper provided by China EAP Association’s official website.^2^ The structure and format of the official sample paper are the same as the actual test paper, and its degree of difficulty is much the same as the actual test paper. Although the number of reading passages in the official sample paper is limited, it is a “sample” that is supposed to reflect the features of the actual test papers. The sample paper consists of two sections: a written test and an oral test. The TEAP corpus consists of six passages, containing a total of 2,958 tokens. The collected test papers of CET and TEAP are all cleaned and manually checked to ensure the accuracy of the calculation.

### 3.2. Instrument and procedure

#### 3.2.1. Compleat lexical tutor

To explore the academic vocabulary coverage of CET-4, CET-6, and TEAP, Academic Word List (AWL) compiled by Coxhead (2000) is employed in this study to analyze the academic word coverage in the English tests under discussion. An online corpus tool Compleat Lexical Tutor,^3^ developed by Professor Tom Cobb of the University of Quebec, is run to calculate the AWL coverage of each text or passage. The tokens of the AWL words and the total tokens of each passage can be obtained from the result page. The total AWL coverage of each test is calculated via the following formula:


$$\mathrm{AWL}\;\mathrm{coverage}=\frac{\mathrm{AWL}\;\mathrm{words'}\;\mathrm{tokens}}{\mathrm{Total tokens}}$$

#### 3.2.2. Flesch–Kincaid Grade Level

Readability refers to the easiness for readers to understand a particular text (Li, 2018), affected by text factors such as lexical, syntactic, semantic, and stylistic complexity, and reader factors such as the reader’s psychology, background knowledge, and personal motivation. Its quantitative measurement could be the lens to estimate the text’s difficulty (Richardson and Morgan, 1997). Readability can be calculated via specific readability formulas such as Flesch Reading Ease, Fog Index, Automated Readability Index, and Grade Level. Without exception, all the above calculations excluded the reader factors, which are difficult to measure or quantify. To figure out the correlation between AWL coverage and language difficulty of the reading passages in the three kinds of tests, the formula Grade Level that has been proven effective and feasible in measuring the difficulty (the opposite of readability) of a text (Walters and Hamrell, 2008) is applied to this study.

Grade Level formula was developed by Professor Rudolph Flesch of Columbia University in 1948. The quantitative results of the Grade Level are marked as 12 different levels. The higher the Grade Level of a text, the more difficult it is for people to read. The 12 levels correspond to the reading level of the average students in the 12 different grades in American schools (Li et al., 2019). In order to discuss the correlation between AWL coverage and language difficulty, the latter is first calculated from the “Readability Statistics” function of Microsoft Word and then analyzed in Microsoft Excel. The formula for calculating the Grade Level is as follows:


(0.39∗ASL)+(11.8∗ASW)−15.59

ASL refers to the average sentence length and ASW is the average number of syllables per word. According to the formula, Grade Level and text difficulty are positively correlated; that is, a higher Grade Level means a more challenging text.

## 4. Results

### 4.1. The AWL coverage of CET and TEAP

The AWL coverage formula is employed to calculate the overall AWL coverage of CET-4, CET-6, and TEAP and the results are shown in Table 2. The results of the three tests are all closer to 10% and farther away from 1.4%. Coxhead (2000, 2011) and her followers suggest that a corpus with an AWL coverage of 10% is indicative of a typical academic corpus. In contrast, a corpus with an AWL coverage of 1.4 is considered a typical corpus of general English. In this sense, the texts of these tests demonstrate, to a lesser degree, the lexical features of academic English.

**Table 2:** The overall AWL coverage of CET-4, CET-6, and TEAP.

Test	AWL tokens	Total tokens	AWL coverage (%)
CET-4	7,090	107,386	6.60
CET-6	9,454	127,143	7.44
TEAP	228	2,958	7.71

As was stated above, CET aims to test whether test-takers possess the necessary skills and knowledge included in the “basic requirements” and “intermediate requirements” outlined in the *Guidelines*. These progressive requirements are reflected, to some extent, in the different academic levels of CET-4 and CET-6, matching their AWL coverage difference: 7.44% (CET-6) > 6.60% (CET-4). In a way, the “basic requirements” can correspond to the objectives of CET-4 and the “intermediate requirements” to those of CET-6. The above data seems to suggest that the academic vocabulary coverage of CET-4 and CET-6 corresponds to the vocabulary requirements of the *Guidelines.*

As was mentioned in Section 2.3, the *Guidelines* proposed different requirements at the basic and the intermediate levels. Chinese college students must acquire an extra 2,000 words on top of the vocabulary they learned in high school to meet the “basic requirements.” Out of these 2,000 words, 400 are crucial in relation to their academic studies or future career prospects. To meet the “intermediate requirements,” which are more demanding, students need to learn an additional 3,000 words. These should also include 600 words that are vital to their academic studies or future jobs (The National Foreign Languages Teaching Advisory Board under the Ministry of Education, 2020). A basic calculation tells us that to meet the “intermediate requirements,” students need to master another 1,000 words after reaching the “basic requirement” and another 200 words related to the academic study and future work.

Distinct from CET, TEAP is a newly developed test to examine students’ oral and written competence in academic English via the academic rating scale and vocabulary stipulated in the *Framework*. According to the syllabus of TEAP, this test is set to measure college students’ English competence in academic research and academic English teaching. Therefore, TEAP is more clearly oriented toward academic English compared with CET, and it makes sense that the AWL coverage of TEAP (7.71%) is higher than that of CET.

#### 4.1.1. The AWL coverage of CET-4 and CET-6 test paper

**Figure 2:**
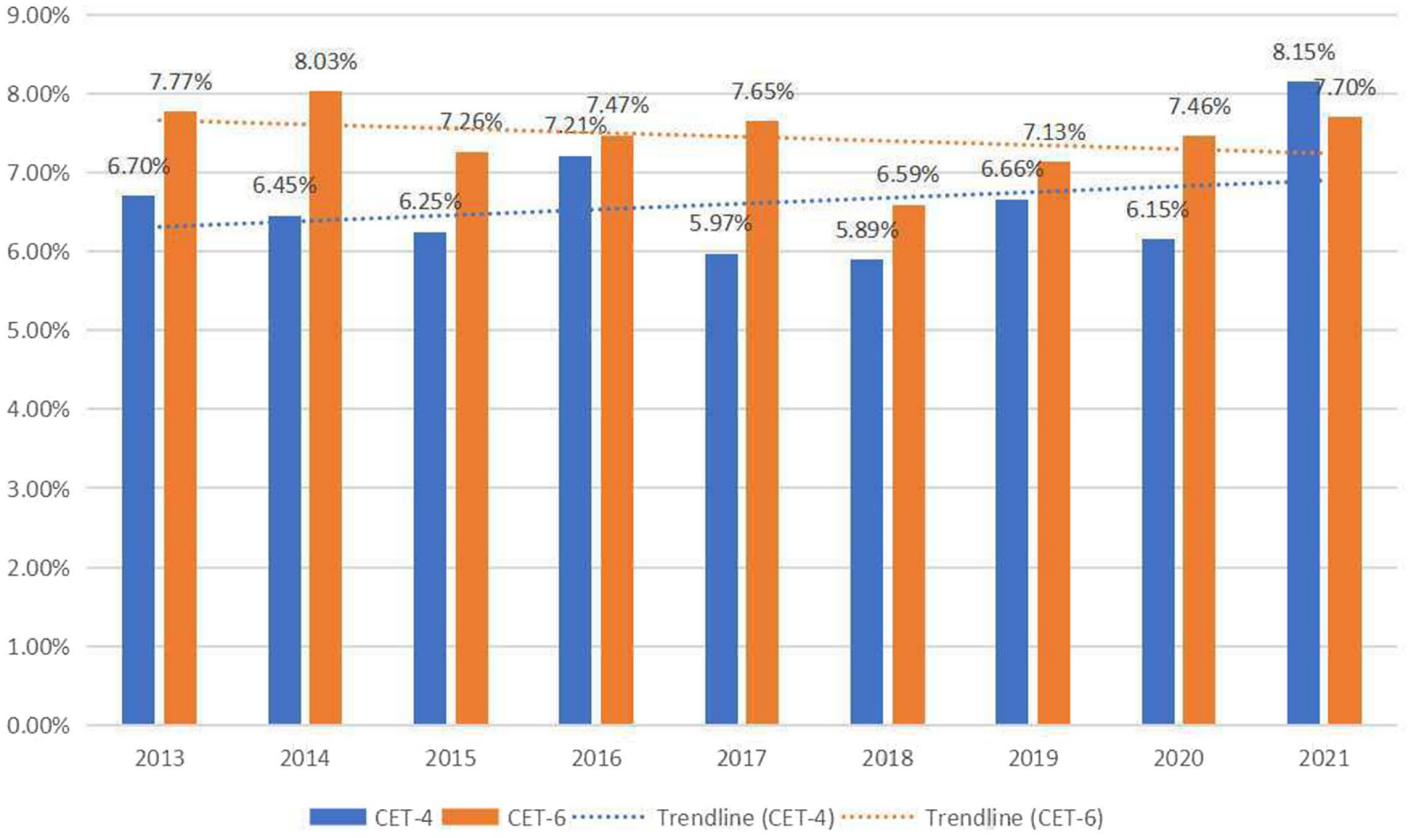
The AWL coverage of CET-4 and CET-6 (2013-2021).

To illustrate the academic feature of CET in the sense of academic language, the AWL coverage from 2013 to 2021 of CET-4 and CET-6, and their trendlines are demonstrated in detail (see Figure 2).

As shown in Figure 2, the AWL coverage of CET-6 is generally higher than that of CET-4. From 2013 to 2021, the AWL coverage of CET-4 fluctuates between 5.5% and 8.5%, and that of CET-6 fluctuates between 6.5% and 8.5%. The fluctuation range is not wide, indicating that the academic nature of CET is relatively stable. It is also shown in Figure 2 that in most of the years, the AWL coverage of CET-6 is higher than that of CET-4.

To have a more scientific description of the difference in academic vocabulary coverage of all passages between CET-4 and CET-6 from 2013 to 2021, an Independent Sample *t* Test is conducted, which serves to compare the means of two independent groups and provides statistical evidence to testify whether the associated population means are significantly different. It is found that there is a significant difference between the AWL coverage of reading passages in CET-4 and CET-6 (see Table 3).

**Table 3:** Independent sample *t* test for CET-4 and CET-6.

	CET-4	CET-6	t(358)	*p*	95% CI
Value	*M* = 0.0669	*M* = 0.0724	1.799	0.0728	−0.00 to −0.01

Figure 2 indicates that there has been a gradual reduction in the difference of AWL coverage between the two tests over the years. This aligns with the incremental standards for CET-4 and CET-6 as outlined in the *Syllabus* and the *Guidelines*. The use of academic vocabulary in the reading materials of both tests shows an attempt to bridge the gap and facilitate the shift from basic to intermediate levels of proficiency.

To have a more comprehensive understanding of the AWL coverage of CET-4 and CET-6, the AWL coverage of each reading passage in CET-4 and CET-6 is also calculated. The results are summarized in Table 4.

**Table 4:** Interval distribution of AWL coverage of reading passages in CET-4 and CET-6.

AWL coverage	CET-4	CET-6
(0%–2%]	1	1
(2%–4%]	34	19
(4%–6%]	50	59
(6%–8%]	61	57
(8%–10%]	36	32
(10%–12%]	16	24
(12%–14%]	3	6
(14%–16%]	3	5
(16%–18%]	0	1
Total	204	204

Table 4 shows the distribution of 204 reading passages in CET-4 and CET-6 respectively; the second and third columns refer to the number of reading passages whose AWL coverage falls in a specific range as the first column indicates. Table 4 shows that the AWL coverage for the majority of reading passages in both CET-4 and CET-6 falls between 4–6% and 6–8%. It implies that most of the materials for CET are neither typical academic English texts nor standard general English texts. Another noticeable finding is that as for passages with AWL coverage falling within the range of (10%–18%], CET-6 has more passages with higher AWL coverage than CET-4, that is, 14 passages. It indicates that the higher band of CET tends to focus more on testing the academic literacy of non-English majors in China. In addition, Table 4 demonstrates that in CET-4 there is only one passage whose AWL coverage is equal to or below 2% and in CET-6 there is also one passage with AWL coverage between 16% and 18% or equal to 18%. Both articles are from the tests in 2014. For CET-4, this article is a narrative essay, where the author narrates the story of racial discrimination against black people in the small town of Piedmont. In general, this article uses few academic words, thus leading to a lower AWL coverage. In contrast, the other article in CET-6 is an argumentative essay, mainly focusing on two issues discussed by investors on US government bonds. The full text involves much academic vocabulary so that the coverage of academic vocabulary is relatively high.

#### 4.1.2. The AWL coverage of TEAP sample test paper

**Figure 3:**
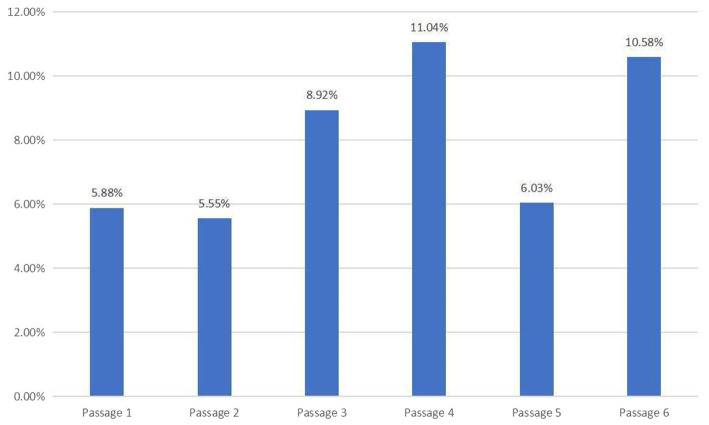
The AWL coverage of six passages in TEAP sample test paper.

Due to the confidentiality and inaccessibility of TEAP test papers, this study analyzed one sample test paper. Even with this small sample, however, it is feasible and reasonable to examine the reading passages in this sample test paper and make some inductions. The AWL tokens, total tokens, and AWL coverage of each passage of the TEAP sample test paper are shown in Table 5, and the AWL coverage is illustrated in Figure 3.

**Table 5:** AWL tokens, total tokens, and AWL coverage of the six passages of TEAP.

Passage	AWL tokens	Total tokens	AWL coverage (%)
1	35	595	5.88
2	38	685	5.55
3	80	897	8.92
4	34	308	11.04
5	12	199	6.03
6	29	274	10.58
Total	228	2,958	7.71

It is noted that the AWL coverage of TEAP fluctuates between 5% and 11%, and within this range, Passages 3, 4, and 6 have comparatively higher AWL coverage. The total AWL coverage of the TEAP sample test paper is 7.71%, slightly higher than that of CET-6 (7.44%).

As an emerging EGAP test, TEAP is supposed to match the standard of academic English tests. However, there are three passages whose AWL coverage is within the range of 5%–6% and only two passages demonstrate AWL coverage above 10.0%.

In the strict sense, Passages 1 and 2 are not typical academic English texts: Passage 1 (5.88%) is excerpted from the essay collection of *The Immediate Experience: Movies, Comics, Theatre & Other Aspects of Popular Culture* (Warshow and Cavell, 2001). This passage mainly discusses the deep insight of western film on the problem of violence. Some of the high-frequency content words in the text, such as values, movies, violence, hero, image, and so on, are not included in Coxhead’s (2000) academic word list and are regarded as general English vocabulary. Passage 2 (5.54%) is an excerpt from the anthology of *The Norton Reader* (Zinsser, 2003). This passage mainly discusses college students in the 1970s facing four kinds of pressure: economic pressure, parental pressure, peer pressure, and self-pressure. The high-frequency content words in the article include college, pressure, student, graduate, and so on. The academic characteristics of the two passages appear unclear, suggesting that they may not be appropriate as reading materials for an academic English test.

Passages 3–6 are all about infants feeding practices selected from academic papers which can be retrieved in Science Citation Index (SCI): Passage 3 (8.92%) is selected from *Food and Nutrition Bulletin* (Anyanwu and Enwonwu, 1985), omitting the parts of Discussion and Recommendation in the original text; the other three passages are all taken from the abstract section of academic papers. Specifically, Passage 4 (11.04%) is from the *Asia Pacific Journal of Clinical Nutrition*, Passage 5 (6.03%) is from *Nutrition,* and Passage 6 (10.58%) is from *Social Science & Medicine*. These passages are more suitable for academic reading comprehension, as evidenced by their sources and the higher AWL coverage. However, the overall AWL coverage of the test is not high, indicating that the academic nature of TEAP needs to be improved. It might be necessary to adjust the AWL coverage of TEAP to further differentiate itself from CET-6.

### 4.2. AWL coverage and language difficulty of College English Tests

As the quantitative measurement of readability, Grade Level has been applied to study the reading comprehension of CET, TOEFL, and IELTS (Li and Yang, 2013; Jiang and Han, 2018), which would give a hint about setting up different levels of testing. A higher Grade Level means a higher cognitive load upon students in understanding those challenging texts. Grade Level can also be analyzed together with AWL coverage. Li et al. (2019) found a significant correlation between AWL coverage and Grade Level of a series of academic textbooks called *New College English*; this suggests that passages in academic textbooks with higher AWL coverage are more difficult to understand.

In order to verify the correlation between AWL coverage and Grade Level in reading passages from CET and TEAP, the Correlation Coefficient (r) of each test is calculated via the CORREL function of Microsoft Excel. According to Cohen (1988), there are three types of correlation: weak (0.1 ≤ |*r*|<0.3), moderate (0.3 ≤ |*r*|<0.5), and strong (0.5 ≤ |*r*|<1). The correlation analysis of CET and TEAP is shown in Table 6.

**Table 6:** Correlation coefficient (*r*) between AWL coverage and grade level of reading passages in three tests.

	CET-4	CET-6	TEAP
*r*	0.496	0.412	0.510

Table 6 demonstrates that the AWL coverage of the reading passages in CET-4 and CET-6 has a moderate correlation with their Grade Level, and that of TEAP has a strong correlation. This suggests that statistically, texts with higher AWL coverage are more challenging to comprehend.

## 5. Discussion

### 5.1. Implications for language testing

With the transition of teaching policy in China from teaching of EGP to incorporating EAP into college English teaching and testing and with the issue of government documents of the *Framework*, the *Syllabus*, and the *Guidelines*, the call for the reform of CET-4 and CET-6 grows increasingly vocal. For many years the debate has been ongoing over the orientation of China’s college English teaching, as was illustrated in the Introduction part of this paper.

So far, some research projects have been conducted to improve the quality of CET. For example, Jin and Yang (2018) profiled a 30-year development of CET and expounded on the direction of language testing with Chinese characteristics. Many scholars discussed different parts of CET, such as listening (Guo, 2013), speaking (Jin et al., 2020), reading (Jiang and Han, 2018), and translation (Lu, 2014). However, very few research endeavours have been made to explore whether CET should be oriented towards test of EGP or EAP.

We believe that before we discuss whether CET should be oriented towards test of EAP, we need to figure out a way to measure whether a test of English is academic in language. In this study, we have calculated the AWL coverage of the reading passages of a test of English to achieve this purpose. In other words, the AWL coverage serves as a quantitative indicator of the academic level of a corpus composed of the reading passages in CET. We find that CET-4 and CET-6 can be considered, to a lesser degree, as tests of EAP in that the AWL coverage of the two types of tests are 6.60% and 7.44% respectively, which is significantly closer to 10% than to 1.4%. This finding is important because it indicates that CET-4 and CET-6 can assess students’ mastery of academic English, as is required in the *Guidelines* and the *Syllabus.*

The study also compares the AWL coverage of TEAP with CET-6. TEAP, as an academic English test, is supposed to score higher in AWL coverage given its clear academic positioning illustrated in the *Framework*, but the total AWL coverage of TEAP (7.71%) is only a little higher than that of CET-6 (7.44%). This demonstrates that the academic level of TEAP is statistically similar to that of CET-6 and that there is no distinct difference between the two types of tests in terms of AWL coverage. Therefore, compared with CET, TEAP cannot better reflect students’ academic level. In addition, TEAP, without sufficient government support, has been suspended for several years, possibly due to the fact that the issue of academic English teaching is still controversial in China. On the one hand, the data from the present study seem to suggest that students’ proficiency in EAP has already been tested nationwide in China for many years despite the dispute on whether EAP should be taught in Chinese colleges and universities. On the other hand, the above data suggests that there seems to be very little need for the present TEAP to be conducted when CET can perform the same function and when the present TEAP does not seem to have performed better in this regard.

In addition, to better differentiate CET-4 from CET-6 in terms of the AWL coverage of the reading passages, the gap in the AWL coverage of the reading passages between CET-4 and CET-6 can be reasonably widened in accordance with the different requirements outlined in the *Syllabus*. For instance, CET-4 can be designed to be more inclined towards the testing of EGP and CET-6 can be designed to be more inclined to the testing of EAP.

This study can also serve as a reference for researchers who aim to explore the academic level of other tests of English. For example, the academic degrees of the international language tests, such as TOEFL and IELTS, remain to be quantified, compared and contrasted based on the calculation of the AWL coverage of these tests.

### 5.2. Implications regarding the correlation between language difficulty and academic level

The last two decades have seen a massive increase in academic exchanges in EAP field (Thompson and Diani, 2015). In this context, academic English has become a necessity for college students.

As was discussed in the Introduction part of this paper, there is a significant debate within the academic community in China regarding whether EGP or EAP should be taught to college students in China. This debate is largely based on whether there exists a relationship between language difficulty and language academicity. This study investigated the relationship between AWL coverage and language difficulty (indicated by Grade Level) in the three English proficiency tests under discussion in this paper, namely CET-4, CET-6, and TEAP. The results showed that there is a positive correlation between AWL coverage and language difficulty in the three tests, with correlation coefficients being 0.496, 0.412, and 0.510, respectively. Statistically, the higher the AWL coverage in a reading passage is, the higher its Grade Level is. In other words, the more academic the language of a reading passage is, the more difficult it tends to be. This study suggests that the correlation between AWL coverage and language difficulty should be considered in language testing design. For instance, if academic English tests are designed for students with lower English proficiency, then the reading passages should not be selected by random sampling. Instead, passages with high AWL coverage but low Grade Level should be selected. To be specific, the reading passages in the test should have an AWL coverage of around 10% (the norm of academic English) and in the meantime feature a Grade Level (language difficulty) lower than that of the randomly sampled materials. This approach will ensure that the reading passages in the test are both academic in language and easy enough to read for these students with lower English proficiency.

Moreover, the results of the present study seem to support Wang and Yao (2013), Wang and Wang (2019) and their followers’ view that EGP should be the main content of college English teaching (and hence testing) and EAP can only be taught in top universities in China where students have a better command of the English language. However, Cai (2012, 2015, 2022) also makes sense in that EAP should be taught to Chinese college students because these students learn English mainly for the academic purpose rather than mainly for general communication. A certain level of academic literacy is required to complete assignments, papers, and future academic research. Thus, it is beneficial for college students to master core academic words (Xodabande et al., 2022). Through mastery of academic words and proficiency in academic reading, students can better obtain academic resources and promote academic communication (Chang et al., 2023). Alshehri and Zhang (2022) have further confirmed the importance of vocabulary size for reading comprehension by focusing on syntactic parsing and semantic association in text comprehension for L2 readers.

But, if EAP is taught to students with lower English proficiency, then the reading passages should not be selected by random sampling. Instead, passages with high AWL coverage but low Grade Level should be selected. To be specific, the reading passages in the textbooks should have an AWL coverage of around 10% (the norm of academic English) and in the meantime feature a Grade Level (language difficulty) lower than that of the randomly sampled materials. This approach will ensure that the reading passages in the textbooks are both academic in language and easy enough to read for these students with lower English proficiency.

## 6. Conclusion

Based on the AWL developed by Coxhead in 2000, this study has used AWL coverage to explore whether China’s tests of English for college students, CET-4, CET-6, and TEAP in particular, are academic in language, and whether these tests meet the requirements of the corresponding government documents, the *Syllabus*, the *Guidelines*, and the *Framework*, to be specific. The study has also examined the correlation between AWL coverage and language difficulty of the reading passages in these tests.

This paper is intended to make three contributions to EAP teaching and testing. First, it has figured out a way to evaluate how academic a text is in language by calculating its AWL coverage. This has enabled us to measure whether a test of English is academic or general in language. This can also make it possible to measure whether the teaching of English is academic or general in language in future research. Secondly, this paper, by using the above method, has calculated the AWL coverage of the reading passages in CET and TEAP, and, in this way, has shown that these two types of tests meet the requirements of the corresponding government documents. The slight difference between CET-6 and TEAP in AWL coverage seems to indicate that CET-6 has the potential to become a test of academic English and that TEAP needs to be re-positioned in order to differentiate itself from CET-6. Thirdly, this paper reveals that language difficulty and academic level of an English text correlate in the statistical sense.

College English Tests have been highlighted recently in the tide of academic reform in China. This study has provided valuable insights into the ongoing debate in China regarding the prioritization of EGP or EAP in college English teaching. Although the corpora in this study are obtained from tests in China, it is reasonable to argue that the method used in this study can also be applied to corpora composed of tests of English in other parts of the world, and even to corpora composed of any texts of English. In this sense, the method used in this study has implications for the research of EAP teaching and testing in the whole world. For example, EAP test designers can enhance the validity of EAP tests by incorporating AWL coverage and Grade Level into their test design. This enables them to make informed decisions when selecting reading comprehension texts, resulting in a more thoughtful and data-driven approach. This will lead to an improvement in the overall quality of EAP tests.

This paper has two limitations. One is that the corpora of this study are not inclusive of everything in the three tests. The CET corpus has only included the reading passages of CET-4 and CET-6 but has excluded the listening, writing and translation parts. The TEAP corpus is representative but very small in size. The other is that this study has relied on AWL coverage to determine whether a text is academic or not, and has excluded other parameters that may be relevant to the academic level of a text in language.

The research scope can be further expanded in two ways. One is that the corpora can be expanded to include other components of a test of English such as listening, writing, and translation parts. The other is that parameters at syntactic, semantic, and discourse levels may be taken into consideration to measure how academic a text is.

## Data availability statement

The original contributions presented in the study are included in the article/Supplementary material, further inquiries can be directed to the corresponding author.

## Author contributions

HiL conceptualizes and drafts the manuscript, designs the research methods and procedures, conducts the data analysis, and proofreads the manuscript. XS sorts out the relevant literature and writes the literature review, builds the corpora, collects data, and analyses the results. JQ sorts out the relevant literature, collects, and analyzes the data. YS collects and analyzes the data. YH checks the data and proofreads the manuscript. LZ proofreads the manuscript. CY checks the data. HaL proofreads the manuscript and facilitates submission of the manuscript. All authors contributed to the article and approved the submitted version.

## Funding

This work was supported by the National Social Science Fund of China (17BYY103 and 21BYY017).

## Conflict of interest

The authors declare that the research was conducted in the absence of any commercial or financial relationships that could be construed as a potential conflict of interest.

## Publisher’s note

All claims expressed in this article are solely those of the authors and do not necessarily represent those of their affiliated organizations, or those of the publisher, the editors and the reviewers. Any product that may be evaluated in this article, or claim that may be made by its manufacturer, is not guaranteed or endorsed by the publisher.

## Footnotes

^1^College English is an English curriculum for non-English majors in China. It consists of compulsory courses and optional courses.

^2^ http://www.ceapa.cn/d.asp?id=184

^3^ https://www.lextutor.ca/

## References

[ref1] Abdollahzadeh E. Amini F. M. Zandi M. (2022). The relationship between L2 motivation and transformative engagement in academic reading among EAP learners: implications for reading self-regulation. Front. Psychol. 13:944650. doi: 10.3389/fpsyg.2022.944650, PMID: 36248441 PMC9558267

[ref2] Alshehri M. Zhang D. B. (2022). Word-to-text integration components in second language (L2) reading comprehension. Front. Educ. 7:926663. doi: 10.3389/feduc.2022.926663

[ref3] Anyanwu R. C. Enwonwu C. O. (1985). The impact of urbanization and socioeconomic status on infant feeding practices in Lagos. Nigeria. Food Nutr. Bull. 7, 33–37. doi: 10.1177/156482658500700

[ref4] Bachman L. F. (1990). Fundamental considerations in language testing. Oxford: Oxford University Press.

[ref5] Cai J. G. (2012). Testing English for general or specific purposes—a study on the re-orientation of College English Test. Technol. Enhanc. Foreign Lang. 146, 27–32.

[ref6] Cai J. G. (2015). The orientation of college English teaching revisited: EGP and ESP. J. Zhejiang Univ. (Hum. Soc. Sci.) 45, 83–93. doi: 10.3785/j.issn.1008-942X.2015.02.261

[ref7] Cai J. G. (2022). College English as general education or discipline-specific education: the orientation of college English teaching to ESP revisited. Contemp. Foreign Lang. Stud. 03, 84–91. doi: 10.3969/j.issn.1674-8921.2022.03.010

[ref8] Chang L. Y. Wang Y. J. Liu J. Feng Y. Zhang X. Y. (2023). Study on factors influencing college students’ digital academic reading behavior. Front. Psychol. 13:1007247. doi: 10.3389/fpsyg.2022.1007247, PMID: 36710817 PMC9877342

[ref9] Chen Q. Ge G. C. (2007). A corpus-based lexical study on frequency and distribution of Coxhead’s AWL word families in medical research articles. Engl. Specif. Purp. 26, 502–514. doi: 10.1016/j.esp.2007.04.003

[ref10] Cheng A. (2016). “EAP at the tertiary level in China: challenges and possibilities” in The Routledge handbook of English for academic purposes. eds. Hyland K. Shaw P. (New York and London: Routledge), 97–108.

[ref11] China’s College English Test Examination Board (2016). National College English Test syllabus. Shanghai: Shanghai Jiaotong University Press.

[ref12] Cohen J. (1988). Statistical power analysis for the behavioral sciences (2nd ed.). Hillsdale, NJ: Lawrence Erlbaum Associates.

[ref13] Cons A. M. (2012). The use and misuse of academic words in writing: analyzing the writing of secondary English learners and redesignated learners. TESOL J. 3, 610–638. doi: 10.1002/tesj.36

[ref14] Coxhead A. (2000). A new academic word list. Tesol. Quart. 34, 213–238. doi: 10.2307/3587951

[ref15] Coxhead A. (2011). The academic word list 10 years on: research and teaching implications. Tesol. Quart. 45, 355–362. doi: 10.5054/tq.2011.254528

[ref16] Coxhead A. (2012). Academic vocabulary, writing, and English for academic purposes: perspectives from second language learners. RELC J. 43, 137–145. doi: 10.1177/0033688212439323

[ref17] Cribb V. M. Wang X. (2019). Making academic vocabulary count through strategic deployment in oral presentations by Chinese students of English. Lang. Learn. J. 49, 251–264. doi: 10.1080/09571736.2019.1566396

[ref18] Csomay E. Prades A. (2018). Academic vocabulary in ESL student papers: a corpus-based study. J. Engl. Acad. Purp. 33, 100–118. doi: 10.1016/j.jeap.2018.02.003

[ref19] Dong L. Z. (2020). The validity research on reading passages of English test for international communication-a comparative analysis of the validity of the reading test in the Australian occupational English test. Foreign Lang. Test. Teach. 36, 13–20.

[ref20] Douglas D. (2000). Assessing language for specific purposes: theory and practice. Cambridge: Cambridge University Press.

[ref22] Fu D. A. Zhao C. H. (2017). A study of college ESP test in local undergraduate colleges and universities. J. Jiangxi Norm. Univ. (Philos. Soc. Sci. Ed.) 50, 132–137.

[ref23] Gao Y. Bartlett B. (2014). “Opportunities and challenges for negotiating appropriate EAP in China” in English for academic purposes (EAP) in Asia: negotiating appropriate practices in a global context. eds. Liyanage I. Walker T. (Rotterdam: Sense Publishers), 13–32.

[ref24] Gao Y. Cui Y. Q. (2021). To arrive where you are: a metaphorical analysis of teacher identity change in EAP reform. Teach. Teach. Educ. 104:103374. doi: 10.1016/j.tate.2021.103374

[ref25] Guo D. D. (2013). An empirical study on college English listening teaching under the pattern of internet-based CET. J. Jiangsu Norm. Univ. (Philos. Soc. Sci. Ed.), 4, 63–65. doi: 10.16095/j.cnki.cn32-1833/c.2013.s1.001

[ref26] Hu K. B. Xie L. X. (2014). Study on the future development of college English teaching in China. Foreign Lang. World 03, 12–19+36.

[ref27] Hutchinson T. Waters A. (1987). English for specific purposes: the role of the ESP teacher. New York: Cambridge University Press.

[ref28] Hyland K. Tse P. (2007). Is there an “academic vocabulary”? Tesol. Quart. 41, 235–253. doi: 10.1002/j.1545-7249.2007.tb00058.x

[ref29] Jiang J. L. Han B. C. (2018). A study of reading text difficulty of CET6, TOEFL, and IELTS based on Coh-Metrix. Foreign Lang. China 15, 86–95. doi: 10.13564/j.cnki.issn.1672-9382.2018.03.017

[ref30] Jiang X. Song B. B. Jiang Y. Zhai Y. Y. (2020). A study on the readability of reading test texts in Chinese proficiency test (HSK). J. China Examinations 12, 30–37. doi: 10.19360/j.cnki.11-3303/g4.2020.12.005

[ref31] Jin Y. (2004). College English Test on the road of reform. Foreign Lang. China 1, 27–29. doi: 10.13564/j.cnki.issn.1672-9382.2004.01.012

[ref32] Jin Y. (2006). On the improvement of test validity and test washback-the CET washback study. Foreign Lang. World 06, 65–73.

[ref33] Jin Y. Wang W. Zhang X. Y. Zhao Y. H. (2020). A preliminary investigation of the scoring validity of the CET-SET automated scoring system. J. China Examinations 07, 25–33. doi: 10.19360/j.cnki.11-3303/g4.2020.07.004

[ref34] Jin Y. Yang H. Z. (2018). Developing language tests with Chinese characteristics: implication from three decades of the College English Test. Foreign Lang. World 02, 29–39.

[ref35] Jordan R. R. (1997). English for academic purposes. Cambridge: Cambridge University Press.

[ref36] Li Y. (2018). A comparison of TOEFL iBT and IELTS reading tests. Open J. Soc. Sci. 06, 283–309. doi: 10.4236/jss.2018.68023

[ref37] Li H. D. Jiang H. H. (2022). Academic word coverage and grade level of college English textbooks. J. Hangzhou Dianzi Univ. (Soc. Sci.) 18, 59–65. doi: 10.13954/j.cnki.hduss.2022.06.008

[ref38] Li H. D. Wu N. Du Y. Y. (2019). A study on AWL coverage of college English textbooks: taking new college English as an example. J. Hangzhou Dianzi Univ. (Soc. Sci.) 78, 69–73. doi: 10.13954/j.cnki.hduss.2019.05.013

[ref39] Li Y. P. Yang T. J. (2013). Research on relevance and trend of difficulty of traditional reading tests in CET-4. J. Guangxi Norm. Univ. (Soc. Sci.) 6, 124–128. doi: 10.16088/j.issn.1001-6597.2013.06.012

[ref40] Liang L. W. Wang X. M. (2020). On paradigm evolution of academic English literacies in foreign countries in the past fifty years. Foreign Lang. World 05, 55–62.

[ref41] Liu X. H. Gu X. D. (2013). A review of empirical washback studies worldwide over the past two decades. Foreign Lang. Test. Teaching 01, 4–17.

[ref42] Lu Z. F. (2014). From "teaching translation for language training "to "translation pedagogy"-with examples from a new College English Test design. Shanghai J. Transl. 02, 72–74.

[ref43] Martinez I. A. Beck S. C. Panza C. B. (2009). Academic vocabulary in agriculture research articles. Engl. Specif. Purp. 28, 183–198. doi: 10.1016/j.esp.2009.04.003

[ref44] Masrai A. Milton J. (2018). Measuring the contribution of academic and general vocabulary knowledge to learners' academic achievement. J. Engl. Acad. Purp. 31, 44–57. doi: 10.1016/j.jeap.2017.12.006

[ref45] Mu C. J. Zhang L. J. Ehrich J. Hong H. Q. (2015). The use of metadiscourse for knowledge construction in Chinese and English research articles. J. Engl. Acad. Purp. 20, 135–148. doi: 10.1016/j.jeap.2015.09.003

[ref46] Paribakht T. S. Webb S. (2016). The relationship between academic vocabulary coverage and scores on a standardized English proficiency test. J. Engl. Acad. Purp. 21, 131–132. doi: 10.1016/j.jeap.2015.05.009

[ref47] Qin X. B. (2003). ESP: its nature, categories and teaching principles. J. South China Univ. Technol. (Soc. Sci. Ed.) 04, 79–83. doi: 10.19366/j.cnki.1009-055x.2003.04.016

[ref48] Richardson J. S. Morgan R. F. (1997). Reading to learn in the content areas (3rd ed). Upper Saddle River, NJ: Merrill Prentice Hall.

[ref49] Shanghai Foreign Languages Teaching Advisory Board. (2013). A framework of reference for English as a foreign language teaching at tertiary level in Shanghai (trial implementation). Beijing: Higher Education Press.

[ref50] Tang Y. J. Ma X. M. (2020). A study on content validity of source language translation in CET-4 translation testing. Transl. Res. Teaching. 02, 69–76.

[ref51] The National Foreign Languages Teaching Advisory Board under the Ministry of Education. (2015). Guidelines on college English teaching (exposure draft). Beijing: Higher Education Press.

[ref52] The National Foreign Languages Teaching Advisory Board under the Ministry of Education. (2020). Guidelines on college English teaching. Beijing: Higher Education Press.

[ref53] Thompson P. Diani G. (2015). “Introduction” in English for academic purposes: approaches and implications. eds. Thompson P. Diani G. (Cambridge: Cambridge Scholars Publishing)

[ref54] Vongpumivitch V. Huang J. Y. Chang Y. C. (2009). Frequency analysis of the words in the academic word list (AWL) and non-AWL content words in applied linguistics research papers. Engl. Specif. Purp. 28, 33–41. doi: 10.1016/j.esp.2008.08.003

[ref55] Walters K. A. Hamrell M. R. (2008). Consent forms, lower reading levels, and using Flesch-Kincaid readability software. Drug Inf. J. 42, 385–394. doi: 10.1177/009286150804200411

[ref56] Wang H. G. (2012). Validity assessment and language proficiency description of PTE academic. Foreign. Lang. Lit. 28, 130–133.

[ref57] Wang Y. W. (2021). A comparative study on readability of reading texts in coursebooks and testing for English majors. Foreign Lang. Res. 02, 70–75. doi: 10.13978/j.cnki.wyyj.2021.02.010

[ref58] Wang S. R. Wang H. X. (2019). Keeping the right direction and making new changes: promoting the quality development of college foreign language teaching. Foreign Lang. World 02, 7–13.

[ref59] Wang S. R. Yao C. H. (2013). Some thoughts on English for academic purposes (EAP) teaching. Foreign Lang. China 10, 4–10. doi: 10.13564/j.cnki.issn.1672-9382.2013.05.011

[ref60] Ward J. (2009). A basic engineering English word list for less proficient foundation engineering undergraduates. Engl. Specif. Purp. 28, 170–182. doi: 10.1016/j.esp.2009.04.001

[ref61] Warshow R. Cavell S. (2001). The immediate experience: movies, comics, theatre & other aspects of popular culture. Cambridge: Harvard University Press.

[ref62] Xi X. M. Zhang C. Q. (2020). Validity and validation in language assessment: development and challenges. J. China Examinations 06, 19–26. doi: 10.19360/j.cnki.11-3303/g4.2020.06.004

[ref63] Xodabande I. Iravi Y. Mansouri B. Matinparsa H. (2022). Teaching academic words with digital flashcards: investigating the effectiveness of mobile-assisted vocabulary learning for university students. Front. Psychol. 13:893821. doi: 10.3389/fpsyg.2022.893821, PMID: 35774936 PMC9239377

[ref64] Yang H. Z. (2018). English for academic purposes or general English? Foreign Lang. World 05, 27–33.

[ref65] Yang H. Z. Weir C. (1998). Validity research on CET-4 and CET-6. Shanghai: Shanghai Foreign Language Education Press.

[ref66] Yapp D. De Graaff R. Van Den Bergh H. (2021). Effects of reading strategy instruction in English as a second language on students’ academic reading comprehension. Lang. Teach. Res. 1-24:136216882098523. doi: 10.1177/1362168820985236

[ref67] Zhang Z. B. (2003). Discussion on teaching reform of foreign language in China. J. Foreign Lang. 04, 1–6.

[ref68] Zinsser W. (2003) in College pressures [1979]. eds. Peterson L. H. Brereton J. C. (New York: Norton)

[ref69] Zou S. (2007). Test specifications and reading assessment-designing the new TEM-4 reading test specifications. Teaching Engl. China 30, 3–15.

